# Validation of the Greek version of the Affiliate Stigma Scale among mothers of children with autism spectrum disorder

**DOI:** 10.1192/bjo.2021.1083

**Published:** 2022-01-20

**Authors:** Angelos Papadopoulos, Dionysios Tafiadis, Angeliki Tsapara, Petros Skapinakis, Meropi Tzoufi, Vassiliki Siafaka

**Affiliations:** Faculty of Medicine, School of Health Sciences, University of Ioannina, Greece; Department of Speech and Language Therapy, School of Health Sciences, University of Ioannina, Greece; Department of Speech and Language Therapy, School of Health Sciences, University of Ioannina, Greece; Faculty of Medicine, School of Health Sciences, University of Patras, Greece; Faculty of Medicine, School of Health Sciences, University of Ioannina, Greece; Faculty of Medicine, School of Health Sciences, University of Ioannina, Greece

**Keywords:** Affiliate stigma, validation, Greek version, family caregivers, autism spectrum disorders

## Abstract

**Background:**

Caregivers of children with autism spectrum disorder (ASD) are sensitive to the internalisation of the stigma, known as affiliate stigma, resulting in reduced self-esteem, isolation and poor psychological well-being.

**Aims:**

This study aims to validate the Greek version of the Affiliate Stigma Scale (ASS) among mothers of children with ASD.

**Method:**

The translated version of ASS in Greek was administered to 53 mothers of children newly diagnosed with ASD in two time periods: 1–6 months from diagnosis (time point 1) and 12 months from the initial assessment (time point 2). The control group consisted of 62 mothers of typically developing children.

**Results:**

The ASS total mean score revealed a moderate level of stigma to the ASD group in both assessments. The reliability measures by item showed a satisfactory composite reliability (affective 0.828, cognitive 0.833, behaviour 0.857). Cronbach's alpha revealed that the estimated internal consistency was excellent (*α* = 0.888), and it found a high positive item-total correlation. Receiver operating characteristic analysis results indicated a statistically significant positive discrimination (area under the curve 0.849, *P* = 0.000) between the groups. The cut-off point was 31.00, with a sensitivity of 0.849 and a 1 – sensitivity of 0.258.

**Conclusions:**

The proposed version of the ASS has good psychometric properties and is valid and reliable for measuring affiliate stigma among caregivers of children with ASD in Greece. Health professionals can use it to assess and understand the stigma experienced by caregivers of children with ASD, and design appropriate interventions to reduce their affiliate stigma.

Autism spectrum disorders (ASDs) are the most common neurodevelopmental disorders. The prevalence rate was 1 in 88 children by 2008.^1^ In 2014, the latest data showed that the overall prevalence of ASD was 16.8 per 1000 (1 in 59) 8-year-old children. Boys were four times more likely than girls to be diagnosed with ASD.^2^ In Greece, the data regarding 10- to 11-year-old children (born in 2008 and 2009) reported 2108 ASD diagnoses, with an overall prevalence of 1.15% at the national level (1.18% and 1.13% in 2008 and 2009, respectively^3^). According to the American Psychiatric Association,^4^ children with ASD display deficits in social interaction, exhibit stereotyped and repetitive behaviours, and show marked delay in developing verbal language accompanied by significant disorders in communication.^5^

Having a family member with autism is a huge daily challenge for the family. Parents caring for children with autism report more stress and generally poorer mental health than parents of typically developing children, even of those with other chronic disorders such as chronic illness or behavioural and/or mood disorders.^6,7^ Furthermore, it is well-documented^8,9^ that family caregivers with heavy care burden are sensitive to stigmatisation and, more specifically, affiliate stigma has been reported to be high in caregivers of children with ASD, with negative consequences.^10^ Over the past decade, researchers have formulated various definitions of stigma to better understand its complex and multifaceted effects on well-being.^11^ Erving Goffman^12^ gave the most established definition of stigma in 1963, in his book entitled *Notes on the Management of Spoiled Identity*. Goffman pointed out that stigma refers to traits that are deeply degrading – always according to the current social criteria – and to undesirable forms of diversity so decisive for the kind of social identity attributed to the individual that they are considered as a person with a ‘spoiled identity’.^12^ Link and Phelan^13^ mentioned that stigma consists of five different elements that define and shape the original meaning: labelling, stereotypes (negative beliefs), separation, loss of prestige, and distinctions (behavioural reactions).^14^ Stigmatisation experiences can affect many areas of people's lives, such as the distribution of job and housing opportunities and the provision of health services, as well as a variety of activities in daily life (e.g. entertainment) that define quality of life.

## Affiliate stigma

A large body of literature refers to various types of categorisation of stigma. Three basic types of stigma are often described: public stigma, self-stigma and affiliate or relationship stigma.^15–17^

Specifically, public stigma refers to the negative reactions from society to stigmatised people (e.g. negative judgement about a group, such as regarding their dangerousness, incompetence, etc. with the behaviour response of avoidance and marginalisation).^17^ When these negative public reactions are internalised by people exposed to the stigma, it is called self- stigma. The close relationship that caregivers and family members have with a stigmatised person can make them experience stigma and, as a result, they may develop affiliate stigma, with important negative effect.^17^ In other words, affiliate stigma refers to people who have close friendships or family ties with people stigmatised because of a disability or physical or mental illness.^17^ The caregiver may experience negative self-evaluation, shame, guilt, depression and anxiety, and may withdraw and isolate themselves so that the relationship with the stigmatised person does not become apparent in their social environment.^16^ Additionally, family caregivers may have limited opportunities for positive social interaction, both for themselves and the family member with the disorder, and for activities and services designed specifically for people with this disorder and their families.

## Autism spectrum disorders and stigma

Regarding affiliate stigma among family caregivers of children of ASD, our research was focused on two concepts: parental experience of marginalisation and parental psychological well-being.^18^ Studies indicate that affiliate stigma is a significant predictor of psychological distress in family caregivers of children with ASD,^19,20^ as they experience feelings of shame, reduced self-esteem, embarrassment, sense of inferiority, guilt, fear and marginalisation by their community and relatives.^18^

Moreover, it was found that stigma is related to higher parental stress and lower family quality of life.^18^ Research shows that the stigma experienced by people diagnosed with ASD and their families results from three main characteristics of the disorder. First, autism is considered by certain people as a ‘hidden’ disability, which means that most people with autism do not appear to have a disability until they exhibit behaviour that is considered as deviant by society. Second, some symptoms of autism include socially unacceptable behaviours, such as verbal and behavioural outbursts. Third, people with autism appear to be physically healthy but at the same time suffer from a pervasive disability.^21^ Furthermore, affiliate stigma is, in some cases, the result of attitudes and behaviours based on the following inaccurate beliefs: that parents are to blame for the onset of autism and cannot perform their parental role.^22^

To the best of our knowledge, the study of stigma in families of children with ASD in Greece is limited. A recent qualitative study showed that about half of the parents of children (aged 7–17 years) with autism reported that they had experienced negative stereotypes or prejudices.^23^ However, there is a lack of studies to assess the stigma experienced by Greek parents with the use of validated questionnaires. The recognition and understanding of these experiences, and especially the possible barriers to the caregiving behaviour, as well as the needs and the challenges of parents of children with autism, will significantly contribute to the design and implementation of interventions for both the prevention and management of affiliate stigma, to improve the psychological well-being of all the family members.

In the present study, the sample comprised mothers because they were the main caregivers of the study children with ASD. It is generally known that women are more likely to be the primary caregiver of a person with a physical or mental illness. Women spend more time with family, and caregiving is central to their identity, a behaviour that is greatly reinforced by most societies. Women take on more caregiving activities, report more care recipient difficulties and experience greater distress as a result of caregiving than male carers.^24^

One of the most widely used tools for the assessment of affiliate stigma is the Affiliate Stigma Scale (ASS),^17^ which was developed based on cognitive and behavioural theory to assess the self-stigma experienced by the person caring for a family member with a mental illness or disability. The scale has been administered in many countries, such as Malaysia,^25^ China,^17^ Israel,^18^ Persia^26^ and India,^27^ and in different populations, including caregivers of people with various disorders such as dementia, intellectual disability and mental illness. It has demonstrated good psychometric properties, but has not been validated in the Greek population.

For the aforementioned reasons, the Greek-language adaptation of a scale for measuring stigma in this specific population is crucial.

## Method

### Study design, participants and procedures

This study was performed over a 20-month period. The sample consisted of 53 mothers of children with newly diagnosed ASD (ASD group), and a total of 62 mothers of typically developing children (control group) consented to participate in the study. Caregivers of newly diagnosed children with autism are a particularly vulnerable group, as they may not yet fully understand autism and the new requirements/necessities of enhanced care. Moreover, they probably have not overcome the shock of the diagnosis because they may not have been psychologically prepared.

The translated version of ASS in Greek was administered to the study sample in two time periods: 1–6 months from diagnosis (time point 1) and 12 months from the initial assessment (time point 2). Our aim was to investigate the stigma experienced by mothers in the initial period after diagnosis and 1 year later. Acquiring parental identity is a reflexive process, as raising a child requires the coordination of parenting skills to the constantly evolving needs of the child.^28^ Thus, we assumed that a year after the diagnosis, mothers would have better understood the characteristics of autism, processed the new parental identity of the ‘parent of a child with autism’ and participated in intervention programmes by specialised health services. Therefore, we considered that they would perhaps be able to recognise stigma experiences in a different way compared with the initial post-diagnosis phase.

Regarding the recruitment of the sample, an open invitation was made to join the study (via posters and social media) to certain specialised therapeutic centres in different regions in Greece. The mothers of children with ASD were recruited from private speech therapy centres, occupational therapy centres and a Greek public general children's hospital, where their children were cared for. If they met the inclusion criteria, they were informed as part of the clinical procedure. The inclusion criteria were as follows: new (within a period of 6 months) diagnosis of ASD in the child, absence of other family members with a disability, ability of the mother to understand and complete the questionnaires, and provision of direct care to the child. Of the 58 cases that met the inclusion criteria, two mothers declined to participate in the study, and three cases were excluded from the analysis because of missing data. All of the children whose mothers participated in this study were already involved in early speech therapy and occupational therapy intervention programmes. Participants of the control group had the same inclusion criteria as the ASD group and a similar age range of the children, but no diagnosis of autism. Furthermore, the recruitment occurred after an open call to the community via social media. Moreover, the typically developing children were first checked by a paediatrician. All participants were informed about the nature, purpose and utilisation of the results of this study, and written informed consent was obtained. The study was conducted in accordance with ethical standards as formulated in the World Medical Association Helsinki Declaration (2002), and institutional review board approval was received by the Scientific Committee of Karamandanio Children's Hospital, Patras, Achaia, Greece (approval number: 4173). Full written consent was obtained from the participants before the study, and the protection of the privacy of participants and the confidentiality of the data were ensured.

### Instrument

The ASS contains 22 items and three domains: cognitive (seven items regarding the negative thoughts associated with having a close relationship with the stigmatised person), affective (seven items concerning negative emotions associated with the internalised stigma) and behavioural (eight items about behaviour or actions related to having internalised stigma). The responses are given on a four-point Likert scale ranging from 1 (strongly disagree) to 4 (strongly agree). Higher scores indicate greater levels of affiliate self-stigma. It is short, easy to use and suitable for assessing stigma in a wide range of family caregivers, including children, spouses, grandchildren and other relatives. The English version of the ASS demonstrated excellent psychometric characteristics (Cronbach's *α* = –0.94).^17^ Additionally, the Indian validation^27^ obtained high Cronbach's alpha coefficient values for affective (*α* = 0.87), behavioural (*α* = 0.90) and cognitive (*α* = 0.89) domains, as well as the full scale (*α* = 0.93). Furthermore, the internal consistency of the Malay version^25^ (Cronbach's *α* = 0.92) and Persian version^26^ (Cronbach's *α* = 0.88–0.94) was similar to the Cronbach's alpha for the original version of ASS.^17^

### Instrument translation and adaptation

The translation and adaptation of the ASS in Greek were carried out according to the guidelines set by the minimal translation criteria from the Scientific Advisory Committee (SAC) of the Medical Outcomes Trust.^29^ The minimal translation criteria are outlined as follows. The ASS was assigned to one Greek speech-language pathologist, one Greek psychologist and one linguist, who were very proficient in English. A new version was developed, and a professional bilingual translator back-translated the version into the English language. The back-translation was reviewed, and cognitive debriefing procedures were performed. The term mental illness/intellectual disability was replaced with ASD. The final version of ASS was submitted to a pilot study.

### Statistical analysis

SPSS version 23 for Windows was used to analyse the data. Descriptive statistics included sociodemographic characteristics of the participants and the clinical characteristics of the children. The distribution of variables was evaluated with the Kolmogorov–Smirnov and Shapiro–Wilk tests. The study's non-skewed variables were expressed by means and s.d. *t*-Tests were used to compare the ASS mean scores between the two assessments of stigma (time points 1 and 2) and between the ASD and control group. The cut-off value for the ASS was estimated by using receiver operating characteristic (ROC) curve analysis. The internal consistency of the Greek version of the ASS was defined with Cronbach's alpha coefficient; the acceptable value was ≥0.70.

The test–retest reliability was measured with the Pearson *R* coefficient. A value >0.8 reveals an excellent internal consistency. Moreover, the omega coefficient was calculated as additional reliability coefficients of internal consistency. Furthermore, for the external validity of the ASS, Pearson's correlation analysis between the total scores of the Greek version of the ASS and other scales could not be determined because of the lack of different Greek affiliate stigma scales. Finally, the statistical significance was set at *P* < 0.050, and all reported *P*-values were two-tailed.

## Results

### Characteristics of the sample

The ASD sample consisted of 53 mothers with a mean age of 39.08 years and an age range of 31–49 years. The majority were married (79.2%), and in terms of their educational level, 49.1% graduated from high school and 47.2% completed university studies. The majority of the participants (69.8%) stated that the monthly family income was <€1500 (Table 1). Of the children with ASD, 42 were boys (79.2%) and 11 were girls (20.08%). Regarding the ASD severity, 23 children (43.4%) met the criteria for level 2 (according to the DSM-5), requiring substantial support, and 16 (30.2%) met the criteria for level 3, requiring very substantial support.

**Table 1:** Sociodemographic characteristics of the autism spectrum disorder group and control group

	ASD group	Control group
Mother's age, years, mean ± s.d. (range)	39.08 ± 4.43 (31−49)	36.40 ± 3.15 (30−47)
Father's age, years, mean ± s.d. (range)	42.26 ± 6.40 (32−65)	39.42 ± 4.30 (32−53)
Family status, *n* (%)		
Married	42 (79.2)	56 (90.3)
Divorced	9 (17)	6 (9.7)
Unmarried	2 (3.8)	−
Mother's educational level, *n* (%)		
Gymnasium school	2 (3.8)	−
High school	26 (49.1)	28 (45.1)
University degree	25 (47.2)	34 (54.9)
Father's educational level, *n* (%)		
Primary school	1 (1.9)	2 (3.2)
Gymnasium school	2 (3.8)	−
High school	31 (58.5)	34 (54.9)
University degree	19 (35.8)	26.9 (41.9)
Mother's profession, *n* (%)		
Economically inactive	21 (39.6)	16 (25.8)
Farmer	3 (5.7)	−
Private employee	14 (26.4)	20 (32.3)
Civil servant	13 (24.5)	18 (29)
Freelancer	2 (3.8)	8 (12.9)
Father's profession, *n* (%)		
Economically inactive	4 (7.5)	2 (3.2)
Farmer	2 (3.8)	4 (6.5)
Private employee	20 (37.7)	15 (24.2)
Civil servant	9 (17.0)	19 (30.6)
Freelancer	18 (34.0)	22 (35.5)
Monthly family income, *n* (%)		
€0–€400	6 (11.3)	2 (3.2)
€400–€800	15 (28.3)	4 (6.5)
€800–€1500	16 (30.2)	18 (29)
€1500–€2500	10 (18.9)	26 (41.9)
≥€2500	6 (11.3)	12 (19.4)

ASD, autism spectrum disorders.

The control group consisted of 62 mothers, with a mean age of 36.40 years and an age range of 30–47 years. Almost all mothers were married (90.3%). The majority of them had a university degree (54.9%) and stated that the monthly family income was >€1500 (61.3%) (Table 1).

### ASS

At time point 1, the total mean score on ASS was 41.63 (s.d. 9.89) in the ASD group and 28.43 (s.d. 8.54) in the control group. The highest mean score in both groups was in the affective domain (ASD group: mean 16.42, s.d. 4.63; control group: mean 10.09, s.d. 3.56). An independent sample *t*-test was performed to compare the mean scores between the two groups, and a statistically significant difference was observed between the total mean scores (*t*(113) = 7.670, *P* < 0.001). In addition, regarding the domains of the scale, the comparison revealed statistically significant differences between the two groups (cognitive: *t*(113) = 6.006, *P* < 0.001; affective: *t*(113) = 8.253, *P* < 0.001; behaviour: *t*(113) = 5.728, *P* < 0.001) (Table 2).

**Table 2:** Comparisons between the two groups of mean scores on the Affiliate Stigma Scale domains at time point 1

Domains	ASD group, *n* = 53	Control group *n* = 62	*t*-test	*P*-value
	Mean (s.d.)	Mean (s.d.)		
Cognitive	12.32 (3.32)	8.00 (2.98)	6.006	<0.001^1^
Affective	16.42 (4.63)	10.09 (3.56)	8.253	<0.001^1^
Behaviour	12.88 (3.33)	9.55 (2.92)	5.728	<0.001^1^
Stigma Scale total score	41.63 (9.89)	28.43 (8.54)	7.670	<0.001^1^

ASD, autism spectrum disorders.

1 *P* < 0.050.

At time point 2, the total mean score on ASS was 2.45 (95% CI 5.14 to −0.237) lower in the ASD group (mean 39.16, s.d. 7.80). This change was not statistically significant (*t*(52) = 1.830, *P* = 0.073). Likewise, the total mean score of the affective domain of the ASS was 16.42 (s.d. 4.63) at time point 1 and 15.13 (s.d. 3.52) at time point 2, for a change of 1.285 (95% CI 2.59 to −0.237). This change was not statistically significant (*t*(52) = 1.970, *P* = 0.073). Similar results were computed for the cognitive domain mean score at time point 1 (mean 12.32, s.d. 3.32) and time point 2 (mean 11.64, s.d. 3.29), for a change of 0.679 (95% CI 1.74 to −0.384). This change was not statistically significant (*t*(52) = 1.282, *P* = 0.205). Also, the mean score of the behaviour domain was 12.88 (s.d. 3.33) at time point 1 and 12.40 (s.d. 2.64) at time point 2, for a change of 0.491 (95% CI 1.41 to −0.434). This change was not statistically significant (*t*(52) = 1.065, *P* = 0.292) (Table 3).

**Table 3:** Between-group comparison of the mean scores at time points 1 and 2 on the Affiliate Stigma Scale

Domains	ASD group (time point 1), *n* = 53	ASD group (time point 2), *n* = 53	*t*-test	*P*-value
	Mean (s.d.)	Mean (s.d.)		
Cognitive	12.32 (3.32)	11.64 (3.29)	1.282	0.205
Affective	16.42 (4.63)	15.13 (3.52)	1.970	0.073
Behaviour	12.88 (3.33)	12.40 (2.64)	1.065	0.292
Stigma Scale total score	41.63 (9.89)	39.16 (7.80)	1.830	0.073

ASD, autism spectrum disorders.

*P < 0.050.

### ROC analysis

**Fig. 1:**
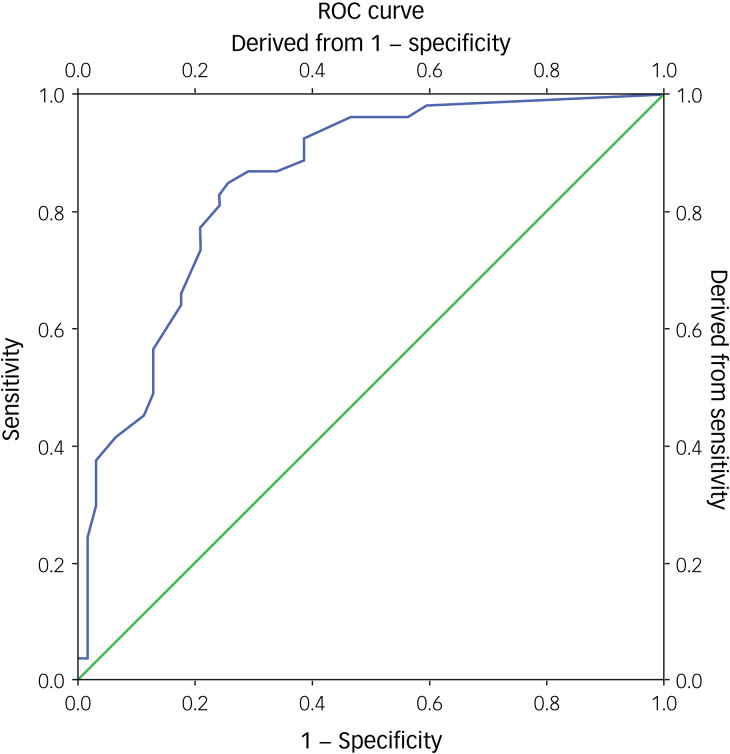
Receiver operating characteristics (ROC) curve of the Affiliate Stigma Scale (ASS) – total score (ASS-T) between the control group and the autism spectrum disorder (ASD) group . The green line represents the reference line and the blue line represents the ROC curve of the ASS-T between the Control group and the ASD Group.

**Fig. 2:**
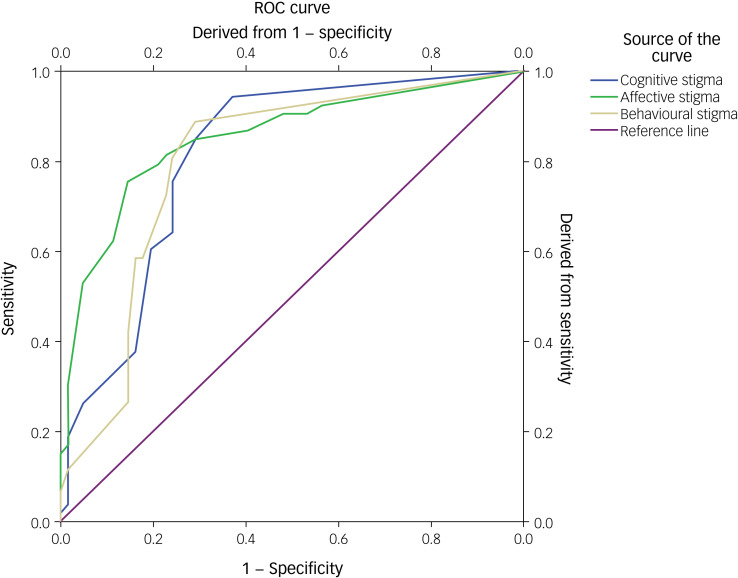
Receiver Operating Characteristics (ROC) curve of the Affiliate Stigma Scale (ASS) Test Subdomains between the Control Group and the autism spectrum disorder (ASD) Group.

Concerning the ROC analysis performed to determine the best possible cut-off points of the ASS total mean score for the ASD group and the control group, the results indicated a statistically significant positive discrimination (area under the curve 0.849, *P* < 0.001) between the groups. Besides, the cut-off point was 31.00, with a sensitivity of 0.849 and a 1 – sensitivity of 0.258 (Fig. 1, Table 4). Also, the ROC analysis revealed statistically significant positive discrimination for all three domains of the ASS. Specifically, for the cognitive domain, the cut-off point was 9.00, with a sensitivity of 0.849 and 1 – sensitivity of 0.0290; for the affective domain, the cut-off point was 15.00, with a sensitivity of 0.623 and 1 – sensitivity of 0.113; and for the behaviour domain, the cut-off point was 11.00, with a sensitivity of 0.717 and 1 – sensitivity of 0.226 (Fig. 2, Table 4).

**Table 4:** Affiliate Stigma Scale receiver operating characteristic data on the discrimination between the autism spectrum disorder and control groups

	Cut-off	Sensitivity	1 – Specificity	AUC (95% CI)	*P*-value
Cognitive domain	9.00	0.849	0.290	0.812 (0.732−0.982)	<0.001^1^
Affective domain	15.00	0.623	0.113	0.853 (0.781−0.925)	<0.001^1^
Behaviour domain	11.00	0.717	0.226	0.798 (0.712−0.883)	<0.001^1^
Stigma Scale total score	31.00	0.849	0.258	0.849 (0.779−0.919)	<0.001^1^

AUC, area under the curve.

1 *P* < 0.050.

### Reliability

Regarding the results of the Cronbach analysis, the estimated internal consistency of the ASS was excellent (Cronbach's *α* = 0.888). Particularly, it found high positive item-total correlation (Table 5). The test–retest reliability of the ASS by item indicated high positive correlations in ASS total mean score (*r* = 0.909, *P* = 0.001) in the cognitive (*r* = 0.965, *P* = 0.001), affective (*r* = 0.936, *P* = 0.001) and behaviour domains (*r* = 0.931, *P* = 0.001) (Table 6).

**Table 5:** Correlation and reliability measures of the Affiliate Stigma Scale by item

Affiliate Stigma Scale	Item-total correlation
Cognitive domain	0.833
Affective domain	0.828
Behaviour domain	0.857

Cronbach's alpha = 0.888.

**Table 6:** Test–retest reliability of the Affiliate Stigma Scale by item

Affiliate Stigma Scale	Pearson's *R* (*P*-value)
Cognitive domain	0.965 (*P* = 0.001)
Affective domain	0.936 (*P* = 0.001)
Behaviour domain	0.931 (*P* = 0.001)
Total score	0.909 (*P* = 0.001)

## Discussion

The present study aimed to culturally adapt and validate the Greek version of the ASS among mothers of children with ASD. Furthermore, the study examined the discriminatory value of the specific scale using a ROC analysis. Based on the findings, the Greek version of the ASS proves to be valid and appropriate, in line with other validation studies.^25–27^

The study group consisted of mothers of children with autism and, more specifically, mothers of newly diagnosed children with autism, covering a research gap in the literature. According to our findings, the mothers of newly diagnosed children with autism reported a low-to-moderate level of affiliate stigma at 1–6 months and 12 months since diagnosis, without significant difference between the two assessments. This result could be explained by the recent diagnosis, which results in the non-accumulation of stigmatising experiences, as well as by the young age of children (mostly preschool age). At this age, the symptoms have not become chronic and the children have not yet been significantly exposed to demanding situations.^30^ Moreover, the recruitment of the ASD group was conducted through institutions that offer various types of interventions (supportive, behavioural therapy, speech and language therapy and occupational therapy), which may have affected the results of affiliate stigma and may explain, at least partially, the low level of stigma.

According to the literature, mothers of children with ASD reported a significant level of affiliate stigma in countries with different cultures,^17,18,20,30,31^ such as China, Hong Kong, India, Israel and six Latin American countries (Brazil, Argentina, Chile, Uruguay, Venezuela and the Dominican Republic). However, it is essential to mention that the role of ethnicity and culture in the stigmatisation procedure is crucial and, for this reason, the comparisons of the findings are not accessible.^31^ It is known that ASD in China differs significantly from ASD in Western countries in terms of prevalence, educational opportunities and other therapeutic interventions, and in general, the course of life of people with autism.^32^ Additionally, Chinese culture is based on collectivism, characterised by a close relationship between individuals and a high sense of obligation to the group. Therefore, children with disabilities can be considered as ‘bad seeds’ and a source of shame for their families.^32^ In Western cultures, other factors, such as the severity of the child's symptoms, may be involved in the stigmatisation process.^18^ In Middle Eastern countries, such as Saudi Arabia, the community and cultural context of understanding autism are complex.^33^ Furthermore, in Saudi Arabia, mothers feel more self-stigma compared with fathers.^34^ Regarding findings from Western cultures, the results of two qualitative studies conducted in Australia^35,36^ showed that parents experienced significant levels of stigma. One of these studies was longitudinal and, at the 10-year follow-up of 28 families of people with autism, found that parents had become less vulnerable to the reactions of others and considered stigmatising behaviours less threatening.^36^ It is important to keep in mind that family caregivers, especially mothers, may perceive their role in caring for their child in positive terms and have positive care experiences. As a result, they may actively resist the negative attitude of others and be less vulnerable to it, having developed a high sense of worth because of the importance of their role.^36^ On the other hand, the literature on the stigmatisation experiences of fathers of children with ASD is limited. However, it seems that fathers experience increased levels of stress to provide the financial resources necessary for the care and future of their children.^37^ In addition, fathers find it more difficult to manage their child's behaviour and, as a result, may be more vulnerable to stigma compared with mothers and avoid participation in social events.^38^

Moreover, in general, adequate and appropriate services are more likely to exist in countries where there is a relatively good level of understanding and awareness about autism. Consequently, in certain cultural contexts, stigmatisation behaviours are expected to be more prevalent.^39^

Regarding the psychometric properties of the ASS, the ROC analysis revealed that the Greek version of ASS showed discriminant validity for measuring normal or stigmatised caregivers. In this study, the calculated cut-off points between the ASD and control group were estimated at 31.00 (area under the curve 0.849) out of a maximum score of 88.00 points. To our knowledge, only the Hindi adaption and psychometric validation of ASS calculated cut-off points derived from the scores’ percentile distribution. Based on the total scores of the obtained data, the 33rd and 66th percentiles were considered the cut-off points to identify low, moderate and high scores on the ASS. The 33rd and 66th percentile are taken as the cut-off point because these two tertile points divide the population into three equal subgroups, and help in categorising them as high, moderate and low scorers.^27^

Furthermore, it should be noticed that the Greek version of the ASS exhibits psychometric properties similar to other studies.^17,25–27,40^ The internal consistency was excellent (Cronbach's *α* = 0.888). Also, the data analysis reported an acceptable range of Cronbach's alpha values of its domains (affective, cognitive, behaviour), and indicated that the items of the Greek ASS are significantly correlated with each other. These findings are in line with the results of other studies.^17,25,27,40^ Additionally, the test and retest reliability of the Greek ASS reported high consistency between the scores and suggested temporal stability. Also, it is consistent with the findings of the Persian and Hindi validation study of the ASS.^26,27^ Therefore, the Greek version of the ASS was found to be a valid scale and can be used as a reliable instrument in clinical practice and future research.

The use of ASS by professionals working with parents of children with ASD is important. Understanding the difficulties and experiences of parents from the reactions of others toward their child and themselves is essential for the design of appropriate and effective interventions, as negative feelings of caregivers can negatively affect children with ASD, exacerbating behavioural problems. For this reason, it is considered necessary to de-stigmatise caregivers of children with autism. Helping parents to self-regulate their emotions should be an essential component of interventions. Cognitive and behavioural therapy techniques, such as cognitive reconstruction, exposure-based behaviour therapies and coping skills training, could also be effective. Interventions should aim to enhance the resilience of parents, providing strategies to prevent and deal with stigma and facilitate their access to services and other sources of social support.^23^ The most effective interventions include psychoeducation of parents about their children's needs and enhancing their self-compassion and conscientiousness.^41^ More specifically, parents should be trained in effective problem-solving techniques, ignoring external negative stimuli, accepting non-critical situations and forgiving their potential faults. Furthermore, it is helpful to encourage parents to focus on the positive aspects of caring for a child with autism and setting aside any negative moments of weakness or self-blaming for the current situation.^41^ In addition, the involvement of parents in self-help and mutual support groups reduces isolation and can significantly contribute to the improvement of their psychological well-being.

### Strengths and limitations

The present study has some limitations. First, the sample is relatively small, and this could weaken the generalisability of the results. However, the sample size is related to our decision to increase sample homogeneity and diagnostic reliability by using a strict selection process, and focus on mothers of newly diagnosed children with ASD. This decision led to a smaller sample that cannot be representative of all of the possible clinical situations. However, with a sample pool of 115 participants, the effect size analysis returned good results for the ASS total score. Although more participants are needed and will be recruited in future research, considering effect size results and limitations, we think that this study's findings can be characterised as a promising start that can lay the foundations for full validation of the instrument in the future. Second, the sample was composed exclusively of female caregivers (mothers), limiting the possibility of generalising the results to male caregivers, as gender differences may be found in stigma experiences.

On the other hand, the main strength of this study is that the ASD sample consisted only of caregivers of newly diagnosed children with ASD. As far as we know, there is a lack of research about the stigma experienced by this group of caregivers.

Despite the above limitations, this study has significant implications for planning interventions for families of children with ASD. As mentioned in detail above, it is necessary to implement interventions to prevent stigma in family caregivers vulnerable to affiliate stigma. Furthermore, it is of paramount importance for the de-stigmatisation of children with ASD, to boost their participation in therapeutic interventions and community activities. Additionally, it is equally important to strengthen the cooperation of health professionals with governmental and non-governmental organisations to provide public education and awareness about ASD, as ignorance is often behind the stigma.^41^ Finally, future research should focus on the study of stigma experienced by other family members (e.g. father, siblings) and examine the possible effects of gender on affiliate stigma. Moreover, further research is needed to determine the mediators in stigma formation in parents of children with autism and the effect of stigma on children's participation in social activities.

In conclusion, the present study examined the translated and adapted in the Greek version of the ASS. The Greek version of ASS is a reliable and valid psychometric tool to measure affiliate stigma among family caregivers with newly diagnosed children with ASD. This scale demonstrated excellent internal consistency, reliability and validity. The statistical results reported in this study agreed with the relevant results of studies on other versions of the ASS across languages and cultures. Finally, this scale can be handy and practical for clinicians and researchers providing a comprehensive evaluation of the affiliate stigma in daily practice.

## Data Availability

The data that support the findings of this study are available from the corresponding author, D.T., upon reasonable request.
